# Microfluidic Design of Ultradeformable Liposomes for Advanced Skin Delivery of *Stellaria media* Phytocomplex

**DOI:** 10.3390/pharmaceutics17111390

**Published:** 2025-10-27

**Authors:** Luigi Ciriolo, Nicola d’Avanzo, Antonia Mancuso, Maria Chiara Cristiano, Antonella Barone, Rosario Mare, Anna Maria Tolomeo, Alexandra I. Comaniciu, Georgiana Nitulescu, Octavian Tudorel Olaru, Felisa Cilurzo, Donatella Paolino, Massimo Fresta

**Affiliations:** 1 Department of Health Science, University of Catanzaro “Magna Græcia”, Viale “S. Venuta s.n.c.”, I-88100 Catanzaro, Italy; luigi.ciriolo@studenti.unicz.it; 2 Department of Experimental and Clinical Medicine, University of Catanzaro “Magna Graecia”, Viale “S. Venuta s.n.c.”, I-88100 Catanzaro, Italy; nicola.davanzo@unicz.it (N.d.); antonia.mancuso@unicz.it (A.M.); barone@unicz.it (A.B.); 3 Research Center “ProHealth Translational Hub”, University of Catanzaro “Magna Graecia”, Viale “S. Venuta s.n.c.”, I-88100 Catanzaro, Italy; 4 Department of Medical and Surgical Sciences, University of Catanzaro “Magna Graecia”, Viale “S. Venuta s.n.c.”, I-88100 Catanzaro, Italy; mchiara.cristiano@unicz.it (M.C.C.); mare@unicz.it (R.M.); 5 Department of Cardiac, Thoracic and Vascular Science and Public Health, University of Padova, I-35128 Padua, Italy; annamaria.tolomeo@unipd.it; 6 Institute of Pediatric Research “Città della Speranza”, I-35127 Padua, Italy; 7 Faculty of Pharmacy, “Carol Davila” University of Medicine and Pharmacy, Traian Vuia 6, 020956 Bucharest, Romania; alexandra.comaniciu@stud.umfcd.ro (A.I.C.); georgiana.nitulescu@umfcd.ro (G.N.); octavian.olaru@umfcd.ro (O.T.O.); 8 Department of Pharmacy, University of Chieti—Pescara “G. d’Annunzio”, Via dei Vestini 31, I-66100 Chieti, Italy; felisa.cilurzo@unich.it

**Keywords:** microfluidic, ultradeformable liposomes, scale-up, antioxidants, *Stellaria media*, dermal delivery

## Abstract

**Background/Objectives:** Ultradeformable liposomes represent an established platform for topical delivery of antioxidant compounds, thanks to their structural flexibility and ability to enhance skin permeation, but standardized manufacturing protocols are still lacking. This study presents a microfluidic-based strategy for the scalable production of ultradeformable liposomes encapsulating *Stellaria media* extract, a polyphenol-rich phytocomplex with strong antioxidant activity. **Methods:** Liposomes were produced with a GMP-like microfluidic platform enabling fine control of formulation parameters and high reproducibility under conditions directly transferable to continuous manufacturing. Process optimization tested different total flow rates. Characterization included particle size and distribution, encapsulation efficiency, colloidal stability and kinetics of release. Permeation was assessed with Franz diffusion cells using human stratum corneum and epidermidis membranes. **Results:** Optimal conditions were a flow rate ratio of 3:1 and a total flow rate of 7 mL/min, yielding ultradeformable liposomes with a mean size of 89 ± 1 nm, a polydispersity index < 0.25, and high encapsulation efficiency (72%). The resulting formulation showed long-term colloidal stability and controlled release. Diffusion studies demonstrated a 2-fold increase in permeation rate compared to the free extract. **Conclusions:** These findings highlight the potential of microfluidics as a robust and scalable technology for the industrial production of ultradeformable liposomes designed to enhance the dermal delivery of bioactive phytocomplex for both pharmaceutical and cosmeceutical applications.

## 1. Introduction

The growing demand for scalable and reproducible nanoscale delivery systems has established microfluidic technology as a key tool for pharmaceutical and cosmeceutical manufacturing. Conventional production methods for lipid-based nanovesicles, such as thin-film hydration or ethanol injection, are limited by batch variability, poor size control, and time-consuming multistep procedures, thus making them unacceptable for industrial translation [1,2]. Microfluidics offers precise control of physicochemical parameters, enabling consistent particle size, narrow and reproducible size distribution, as well as high drug loading under mild conditions [3].

Among lipid-based nanovesicles, liposomes are the first nanomedicines which reached the market in 1995 [4] and still represent a valuable and versatile platform, which can be tailored to optimize the drug delivery through different routes of administration. In these attempts, ultradeformable liposomes demonstrated remarkable potential for dermal delivery due to their high bilayer flexibility [5]. These nanovesicles are made of phospholipids and edge activators and preserve their structural integrity while crossing the intercellular pathways, thus enabling deeper skin penetration and improved efficacy of payloads [6,7,8,9]. Their high biocompatibility and the ability to protect photosensitive phytochemicals make them an attractive candidate for topical delivery of antioxidant compounds [10,11,12,13].

Skin is the largest tissue in our body, and it is exposed to several exogenous and endogenous stimuli which may induce an overproduction of reactive oxygen species (ROS), which increase the skin aging and degeneration rate [14,15]. Chronic inflammations and autoimmune diseases, prolonged exposure to UV radiation, pollutants and lifestyle-related stressors lead to ROS accumulation, overwhelming natural protective mechanisms and causing cumulative cellular damage. Photoaging, manifested by wrinkles, elasticity loss, and uneven pigmentation, is a major consequence of this process [16,17,18]

Natural compounds such as polyphenols, flavonoids, and antioxidant vitamins are particularly attractive as adjuvant of anti-inflammatory therapies, as well as for cosmeceutical applications due to their ROS-scavenging, anti-inflammatory, and tissue-protective features, along with suitable safe profiles and end-user preference for natural ingredients [19,20,21,22]. Among plant sources, *Stellaria media* (L.) Vill. (SM, chickweed) has gained attention for its rich phytochemical composition, including flavonoids, phenolic acids, triterpenoids, alkaloids, and fatty acids [23,24]. Key constituents such as apigenin derivatives, vicenin-2, chlorogenic acid, and ascorbic acid are known for their antioxidant and anti-inflammatory activities, supporting skin protection, hydration, and tissue repair [25]. However, the limited skin permeability of its bioactive compounds severely impairs their topical bioavailability [26,27], thus highlighting the need for advanced drug delivery systems.

In this study, we optimized a good manufacturing practice (GMP)-like microfluidic process to produce ultradeformable liposomes encapsulating SM extract. The optimization of total flow rate (TFR) and aqueous-to-organic flow rate ratio (FRR) allowed the preparation of nanovesicles with ca. 70% of encapsulation efficiency, a mean diameter of 89 ± 1 nm, and a suitable size distribution for dermal application. The resulting formulation also showed proper physical stability by preserving its main physicochemical properties during long-term storage and after lyophilization. SM-loaded ultradeformable liposomes showed a sustained drug release with a two-fold higher permeation rate than free extract through human stratum corneum epidermidis (SCE). These results, along with safety and confirmed antioxidant properties of SM extract, support the potential of the proposed approach for the effective delivery of phytocomplex through intact skin.

## 2. Materials and Methods

### 2.1. Materials

Phosphatidylcholine 94% (PC) (Lipoid S 100) was kindly provided by Lipoid GmbH, Ludwigshafen, Germany. Sodium taurodeoxycholate (STDC), powders of naringin, rutin, neohesperidin, quercetin, apigenin, and hesperidin used as standard molecules, as well as sodium carbonate, sodium nitrite, sodium hydroxide, Folin–Ciocaltêu reagent, methanol (MeOH), acetonitrile (ACN), and 85% phosphoric acid solution were purchased from Merck Sigma-Aldrich (Merck, Darmstadt, Germany). All other used materials and organic solvents were of analytical grade (Carlo Erba, Milan, Italy).

### 2.2. Preparation of Stellaria Extract

The aerial parts of *Stellaria media* (L.) Vill (Caryophyllaceae) were collected during the flowering stage from Orăție Commune, Buzău County, Romania.

The plant material was air dried at room temperature and finely ground prior to extraction. The aqueous extraction, performed with slight modifications of previously described method [28], was carried out using distilled water (1:10 *w*/*v* ratio). The procedure was conducted in round-bottom glass flasks under reflux for 30 min. The resulting extract was vacuum filtered, concentrated under reduced pressure at 40 °C using a rotary evaporator (RVO004, Ingos, Czech Republic), and lyophilized for 24 h at −55 °C using a freeze-dryer (ScanVac Coolsafe, LaboGene, Lynge, Denmark). The final yellow powder, characterized by a distinctive odor, was stored in PVC vials in a desiccator at room temperature.

### 2.3. Characterization of Stellaria Extract

The SM extracts were chemically characterized by different analytical approaches, based on UV-Vis spectrophotometry and liquid chromatography.

Briefly, three colorimetric assays were used for assessing total phenolic content of the extract, as well as to determine their antioxidant activity, according to the methods previously described [29,30]. The analysis was carried out by using a UV-Vis spectrophotometer (ThermoScientific-Genesys^®^ 150, ThermoFisher Scientific, Milan, Italy). The high-performance liquid chromatography (HPLC) was also used for specifically detecting biomolecules contained in the extract, as well as to qualitatively validating the biomolecules released from ultradeformable liposomes.

The extract’ total phenolic content (TPC) was assessed by incubating the sample with Folin–Ciocalteu’s phenol reagent (1:5 *v*/*v* ratio). The resulting mixture was vortexed, incubated for 5 min at room temperature, and then added to 500 µL of sodium carbonate solution. All samples were finally left to react in the dark for 25 min and the absorbance was measured at wavelength (λ) of 760 nm. In this case, gallic acid was used as a reference and TPC was quantified by using a proper external calibration curve (y = 4.804x + 0.006; r^2^ = 0.996) and therefore expressed as milligram equivalents of gallic acid per mL (GAE mg/mL) and GAE mg/g of extract.

The antioxidant potential and free radical scavenging capacity of the extract were evaluated by two distinct colorimetric methods, based on the 2,2-diphenyl-1-picrylhydrazyl (DPPH) and the 2,2′-azino-bis-(3-ethylbenzothiazoline-6-sulfonic acid) (ABTS) radicals, respectively, according to a previously described protocol [30]. For the DPPH assay, 50 µL of each sample were incubated 30 min in darkness with a 0.004% (*w*/*v*) DPPH solution, and the absorbance was subsequently measured at 517 nm. In the ABTS assay, the ABTS radicals were generated by mixing ABTS with potassium persulfate aqueous solution in 9:1 volume ratio, followed by overnight incubation in the dark. The resulting mixture was then diluted to the working concentration, and absorbance was recorded at 734 nm after 5 min incubation with 50 µL of the extract samples. In both assays, L-ascorbic acid (2 mg/mL) was enrolled as a reference antioxidant molecule. Results are expressed as milligrams of ascorbic acid equivalents (EAA) and as percentage of inhibition, the latter calculated according to the following equation:

I (%) = ((A_0_ − A_1_))/A_0_ × 100 (1)

where A_0_ is the absorbance of the negative control (ABTS or DPPH pure solutions) and A_1_ is the absorbance of the extracts or standard solutions.

High-performance liquid chromatography (HPLC) analyses were performed using a ThermoFisher Scientific Vanquish System Base (ThermoFisher Scientific, Rosano, Milan, Italy), equipped with a quaternary pump and a variable-wavelength UV/VIS detector. Data acquisition and processing were carried out with Chromeleon^®^ software (version 7.2). Separation was achieved by using a 100 × 4.6 mm reverse-phase C18 column with 5 µm silica particle size, while the mobile phase consisted of 10 mM H_3_PO_4_ aqueous solution and acetonitrile (ACN). The column temperature was maintained at 30 °C using a forced-air cooling system, and chromatographic signals were monitored at 260 nm and 270 nm. The comparison of peaks’ retention times with those provided by rutin, naringin, hesperidin, neohesperidin, quercetin, and apigenin (listed in decreasing order of hydrophilicity) allowed compound identification in the extracts. Calibration curves were constructed for each reference standard (concentration range 1.56–200 ppm; r^2^ > 0.999 for all compounds; Supplementary Figure S1A) to enable quantitative determination of flavonoids in the samples. A 10 µL aliquot of the extracts was injected for each analysis

### 2.4. Cell Viability Assay

Human dermal fibroblasts (BJ) and keratinocytes (HaCaT) cell lines were used to study in vitro the safety of SM extract. BJ cell line was obtained from ATCC (Manassas, VA, USA) (ATCC^®^ CRL-2522; Cellosaurus ID: CVCL_3653) while HaCaT cell line was provided by Cytion (Cytion, catalog no. 300493; Cellosaurus ID: CVCL_0038). Both cell lines were cultured in Dulbecco’s Modified Eagle Medium (DMEM) under standard conditions. DMEM was supplemented with 1% penicillin–streptomycin and 10% of fetal bovine serum (FBS). BJ and HaCaT cells were seeded into 96-well plates at a density of 5000 and 10,000 cells per well, respectively, and allowed to adhere overnight. The next day, the cells were treated with increasing concentrations of the extract (5, 10, 25, 50, 100, 250, 500, and 1000 µg/mL). Cell viability was assessed after 24 and 48 h of the treatment using the MTT assay. For the assay, 10 µL of MTT solution (5 mg/mL) were added to each well containing 100 µL of culture medium and incubated for 3 h at 37 °C. After incubation, the medium was removed, and the resulting formazan crystals were solubilized with 100 µL of DMSO:EtOH (1:1 *v*/*v* ratio) solution under gently shaking for 20 min to ensure the complete dissolution. Absorbance was measured using a Varioskan™ LUX multimode microplate reader (Thermo Fisher Scientific, WI, USA) at wavelengths appropriate for MTT formazan detection (540 nm with reference at 690 nm).

### 2.5. Scalable Ultradeformable Liposomes Preparation

Ultradeformable liposomes were prepared using the Sunshine Microfluidic System (Unchained Labs, Pleasanton, CA, USA). Briefly, Lipoid S100 and sodium taurodeoxycholate were co-dissolved in ethanol at a total lipid concentration of 20 mg/mL with a molar ratio of approximately 9:1 (PC:STDC) [31]. For formulations containing plant extract, a 10 mg/mL aqueous stock solution was prepared by solubilizing the powder of Stellaria extract in Milli-Q water. Both organic and aqueous phases were sterile filtered through 0.22 µm membranes prior to use.

Ultradeformable liposomes assembly was carried out using a Sunny Trident T 490 microfluidic chip (Unchained Labs, CA, USA). The microfluidic chip used in this study was fabricated in bonded glass using wet-etch lithography and features a trident configuration. Two lateral inlet channels (width 500 µm; depth 490 µm) intersect the central solvent stream at an angle of 45°, forming a focused mixing junction. Downstream of the junction, the channels extend through ten consecutive linear serpentine segments before reaching the outlet (Figure S2).

The system and chip were primed with ethanol and water according to a predefined initialization program. The lipid mixture and aqueous phase were loaded into their respective loops. During the optimization phase, formulations were produced by using different total flow rates (TFRs) while maintaining a constant water-to-organic (W:O) phase flow rate ratio of 3:1 (FRR) (Table 1).

The obtained ultradeformable liposomes were diafiltered using tangential flow filtration (TFF) (Repligen, Waltham, MA, USA), through a 300 kDa mPES membrane (Repligen), with Milli-Q water as the diafiltration medium, to efficiently remove residual ethanol and unencapsulated components.

### 2.6. Physicochemical Characterization

Mean size and size distribution, namely polydispersity index (PdI), of resulting liposomes were studied by using a Zetasizer Nano ZS (Malvern Instruments, Worcestershire, UK). Prior to analysis, samples were diluted with an isotonic solution to minimize multiple scattering effects and transferred into disposable plastic cuvettes. Measurements were performed at a backscattering angle of 173°, using a 670 nm laser diode with a rated output of 4.5 mV. Zeta potential was assessed using the same instrument. Diluted samples were loaded into folded capillary Zeta cells, and measurements were carried out using the Smoluchowski approximation with a F(Ka) value of 1.5.

To confirm the particle size distribution and determine particle concentration, nanoparticle tracking analysis (NTA) was performed using a NanoSight Ultra instrument (Malvern Instruments, Worcestershire, UK), equipped with a 488 nm laser and a high sensitivity sCMOS camera. Samples were diluted in particle-free ultrapure water (filtered through 20 nm Whatman filters) to reach the optimal concentration range (1–10 × 10^8^ particles/mL) recommended for accurate tracking [32]. The diluted samples were introduced into the analysis chamber via a syringe pump operating at a constant flow rate of 3 µL/min throughout the measurement.

### 2.7. Transmission Electron Microscopy

The morphological features of the obtained ultradeformable liposomes were assessed through transmission electron microscopy (TEM), following previously established methods with some modifications [33]. Prior to imaging, the nanovesicles suspensions were properly diluted in an isosmotic buffer to preserve structural integrity. A small aliquot of the diluted sample was carefully deposited onto formvar/carbon-coated copper grids and allowed to adsorb for a few minutes under room conditions. To obtain a suitable contrast, the nanovesicles were negatively stained using a 2% (*w*/*v*) aqueous uranyl acetate solution. The excess staining solution was gently removed, and the grids were air-dried before imaging. TEM micrographs were acquired using a Tecnai G2 transmission electron microscope (FEI Company), operated at an accelerating voltage of 100 kV, and equipped with a Veleta digital camera (Olympus Soft Imaging System). This approach enabled the visualization of nanovesicle morphology and structural integrity at the nanoscale level, providing qualitative confirmation of size, shape, and vesicle integrity.

### 2.8. Entrapment Efficiency and Kinetic Release Profile

The entrapment efficiency of ultradeformable liposomes was indirectly assessed by quantifying the TPC of unentrapped extract. Briefly, the microfluidic-derived sample was subjected to TFF using a 300 kDa mPES membrane (Repligen, USA) with MilliQ water as a diafiltration medium. The process was carried out under continuous flow condition until five diafiltration volumes were reached to enhance removal of unencapsulated molecules. The collected permeate was lyophilized and then reconstituted in Milli-Q water prior to analysis. Encapsulation efficiency (EE%) was calculated indirectly according to the following Equation (2).

EE% = ((P_tot_ − P_out_))/P_tot_ × 100 (2)

where P_tot_ is the TPC measured in the aqueous phase used during the preparation stages and P_out_ is the TPC measured in the filtration permeate.

Drug loading (DL) was calculated as the ratio between the amount of bioactive compound encapsulated within the ultradeformable liposomes and the total weight of the liposomal formulation, expressed as a percentage, according to following equation:

DL (%) = (P_E_/L _tot_) × 100 (3)

where P_E_ is the entrapped amount of extract and L _tot_ is the total amount of raw materials used to prepare SM-ultradeformable liposomes.

The release kinetic profile of the SM-loaded ultradeformable liposomes was evaluated by monitoring the cumulative release of total polyphenols over time. For this purpose, 1 mL of the final formulation was placed into a Midi-Dialyzer^®^ dialysis tube (Millipore, Burlington, MA, USA) with a molecular weight cutoff of 8–10 kDa and incubated under continuous stirring at 37 °C ± 0.5 in 100 mL of Milli-Q water for 8 h. At predetermined time points, 10 mL of the external medium were withdrawn and immediately replaced with an equal volume of fresh Milli-Q water to maintain sink conditions throughout the experiment. The samples collected were then analyzed using the TPC assay and then quantified by using the proper external calibration curve. The SM extract-released percentage (%) was then calculated for each time point by using the following Equation (4) and obtained values were used to construct a cumulative release kinetic curve:

SM Release% = (P_released_/P_loaded_ × d.f.) × 100 (4)

where P_released_ is the amount of released polyphenols, P_loaded_ is the amount of loaded polyphenols, and d.f. is the dilution factor.

### 2.9. Long-Term Stability Studies

The physical and long-term stability of SM-loaded ultradeformable liposomes was assessed through complementary analytical approaches. The colloidal stability was evaluated using a Turbiscan Lab^®^ Expert stability analyzer (Formulaction, L’Union, France). Samples, diluted 1:10 in Milli-Q water to a final volume of 6 mL, were analyzed over their entire height (~10 mm) at 25 °C and 32 °C, simulating storage and physiological conditions, respectively. Turbiscan technology, based on backscattered (BS) and transmitted (T) light detection at 45° and 180°, enabled real-time monitoring of destabilization phenomena such as sedimentation, creaming, flocculation, or aggregation [34]. To reduce interference from air bubbles and glass tube interface, only data between 2.5 and 10 mm were considered. The Turbiscan stability index (TSI) was calculated as a global stability indicator over 1 h of analysis. A variation of BS and T signals below 5% was considered indicative of a stable colloidal formulation. Long-term stability was further studied by storing samples at 4 °C for up to 30 days. At fixed time points (t_0_, 7, 14, 21, and 30 days), hydrodynamic diameter was measured by DLS, while particle concentration (particles/mL) was determined by NTA at t_0_ and after 30 days to detect potential aggregation or vesicle loss. Finally, the impact of lyophilization on colloidal and physicochemical properties was evaluated in the presence and absence of several concentrations of trehalose as a cryoprotectant (2.5%, 5%, and 10% *w*/*v*). Samples were frozen in liquid nitrogen and lyophilized under vacuum (<0.1 mbar) for 24 h. After reconstitution with the starting volume of Milli-Q water, DLS and NTA analyses were performed to assess particle size and recovery rate and detect potential destabilization. To further evaluate the integrity of the liposomes after freeze-drying, the entrapment efficiency of lyophilized formulation was re-quantified. Briefly, samples were reconstituted in Milli-Q water and purified using Amicon^®^ Ultra centrifugal filters (MWCO 50 kDa) to remove the extract leaked during the freeze-drying. The ultrafiltration process was repeated five times, after which the ultrafiltrate was concentrated and analyzed by the total phenolic content (TPC) assay. The extract EE% of lyophilized samples were then indirectly quantified according to Equation (2).

### 2.10. Deformability Assay

The deformability of proposed ultradeformable liposomes was evaluated as described in our previous studies [6]. Briefly, samples were analyzed by DLS to determine their mean size before and after undergoing a controlled extrusion process. Deformability was induced by extruding 1 mL of the formulation through polycarbonate membranes with a pore size of 30 nm (ca. 1/3 of starting nanovesicles diameter [35]), under a constant pressure of 2.5 bar for 10 min at 32 °C. The nanovesicles’ ability to deform itself was inferred from the size variation before and after extrusion, and the recovery weight rate of the sample after extrusion. The deformability index (D.I.) was calculated by using Equation (5).

D.I. = J × (d_0_/p) × (d_0_/(d_0_ − d_1_)) (5)

where J represents the fraction of the formulation recovered following the extrusion process (ranging from 0 to 1), p refers to the pore cut-off of the membrane, while d_0_ and d_1_ correspond to the mean hydrodynamic diameters of the ultradeformable liposomes measured before and after extrusion, respectively.

### 2.11. In Vitro Skin Permeation Studies

The in vitro permeation of natural compounds across the SCE membranes was investigated using Franz diffusion cells (LGA, Berkeley, CA, USA) with a diffusion area of 0.75 cm^2^ and a receptor compartment volume of 6.5 mL. SCE membranes were prepared from human skin samples obtained following the surgical excision of breast tissue from an adult female donor. The isolation procedure was carried out according to previously described protocols [36]. In brief, subcutaneous adipose tissue was gently removed using a surgical scalpel, and the skin was subsequently immersed in distilled water at 60 °C for 2 min. Following this treatment, the SCE layer was carefully separated from the remaining skin structures.

The isolated membranes were then dehydrated by placing them in a desiccator containing calcium chloride. After dehydration, SCE membranes were stored at 4.0 ± 1.0 °C and rehydrated prior to being used in drug permeation studies. The integrity of human SCE membranes was assessed by measuring transepithelial electrical resistance (TEER) prior to initiating the investigation. The measurement on SCE membrane yielded values consistent with previous studies, which demonstrated that TEER values exceeding 60 kΩ cm^2^ were indicative of an intact human stratum corneum [37]. SCE membranes were employed as the barrier between the donor and receptor chambers. A volume of 400 µL of ultradeformable liposomes and 400 µL of free extract (diluted to match the amount of encapsulated extract in the liposomal formulation) were loaded into the donor compartment. The receptor compartment was filled with PBS. At different time points (2 h, 4 h, and 8 h) the entire receptor medium was collected and lyophilized. The resulting powder was reconstituted in Milli-Q water and analyzed using the TPC assay, as previously described.

### 2.12. In Vivo Biosafety on Healthy Human Volunteers

In vivo biosafety of SM-ultradeformable liposomes was assessed on 8 healthy human volunteers with a mean age of 27 ± 9 years. The study was conducted in accordance with the Declaration of Helsinki and the protocols were approved by the Research Ethics Committee of the “Magna Graecia” University of Catanzaro (Ethics approval number 392/2019). Written informed consent was obtained from all participants after receiving full information about the objectives, procedures, and potential risks of participation. All volunteers were acclimatized for 20 min in a temperature- and humidity-controlled environment (24 ± 1 °C; relative humidity 40–50%). Three test sites were delineated on the volar surface of each forearm, maintaining at least 2 cm of separation between them to prevent cross-interference, as previously reported [36]. Treatments included sterile saline solution (0.9% *w*/*v* NaCl), the aqueous SM extract, and the SM-ultradeformable liposomes. A fixed volume of 100 μL was applied to each site with a micropipette.

Cutaneous tolerability and biosafety were evaluated by monitoring multiple biophysical parameters of the skin and the measurements were carried out before (baseline) and after application (1, 2, 4, 6, and 8 h) of treatments. Skin hydration and transepidermal water loss (TEWL) were measured using a C+K Multi Probe Adapter (Courage & Khazaka, Cologne, Germany) equipped with a Corneometer^®^ CM 825 and a Tewameter^®^ TM 300 probe. Melanin and erythema index (EI) were assessed by spectrophotometric evaluation of skin reflectance using an X-Rite Ci62 device (X-Rite Inc., Grandville, MI, USA). The EI, which reflects changes in skin redness, was selected as the primary outcome parameter and was calculated using the following equation:

(6) $$EI=100[log\frac{1}{R560}+1.5\left(log\frac{1}{R540}+log\frac{1}{R580}\right)-2\left(log\frac{1}{R510}+log\frac{1}{R610}\right)]$$

where $\frac{1}{R}$ was the reflectance at specific wavelengths (510, 540, 560, 580, and 610 nm), which correspond to the absorption peaks of hemoglobin and melanin [34].

Melanin values and erythema indices were further confirmed by complementary colorimetric analysis with a Mexameter^®^ MX 18 probe (Courage & Khazaka electronic GmbH, Cologne, Germany).

### 2.13. Statistical Analysis

Statistical analysis performed by using one-way ANOVA test. Analysis was carried out by using SigmaPlot v.12 and Excel (Office 2016) at various significance levels: * *p* < 0.05, ** *p* < 0.01, and *** *p* < 0.001.

## 3. Results and Discussion

### 3.1. Chemical Characterization of SM Extract

**Figure 1:**
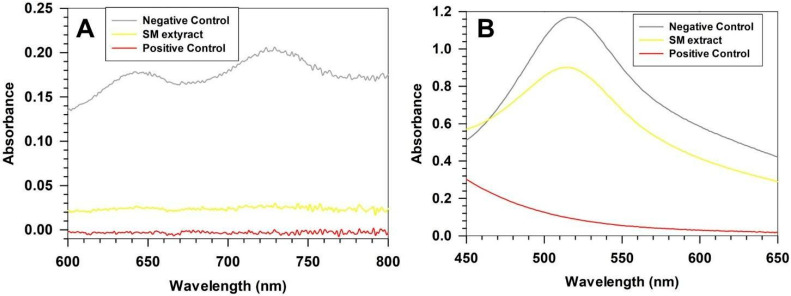
Antioxidant activity of extract. *Stellaria media* antioxidant activity assed by ABTS (**A**) and DPPH (**B**) assay. Curves are representative of three independent analyses ± standard deviation (S.D.).

The chemical characterization of SM extract confirmed a high content of phenolic compounds, consistent with the extract’s notable antioxidant potential (Figure 1 and Table 2).

The TPC was 0.131 ± 0.011 mg/mL gallic acid equivalents (GAE), corresponding to 13.1 ± 1.1 mg of GAE equivalents for each g of extract’s powder (Table 2).

The antioxidant activity confirmed the extract’s marked ability to neutralize hydrophilic radicals, with 84.96 ± 0.71% ABTS inhibition, corresponding to 23.09 ± 0.19 µg ascorbic acid equivalents (AAE) (Figure 1A). In contrast, its lipophilic radical scavenging capacity was lower, as shown by a 23.25 ± 0.14% DPPH inhibition (12.58 ± 0.09 µg AAE) (Figure 1B). These findings demonstrated that the extract predominantly contains hydrophilic antioxidant species.

HPLC analysis further contributed to the characterization of the bioactive compounds within the extract. Among the polyphenols previously quantified by spectrophotometric assays, HPLC specifically identified several flavonoids, such as rutin (0.767 ± 0.011 ppm), naringin (0.216 ± 0.004 ppm), hesperidin (0.226 ± 0.005 ppm), and trace amounts of apigenin (0.018 ± 0.0001 ppm), which are known to play a key role in the antioxidant activity of the extract (Figure S1). Collectively, these data confirm that the SM aqueous extract is rich in phenolic compounds, with significant antioxidant activity, particularly against hydrophilic radicals, supporting its potential for pharmaceutical and cosmeceutical applications.

### 3.2. In Vitro Cell Viability

**Figure 2:**
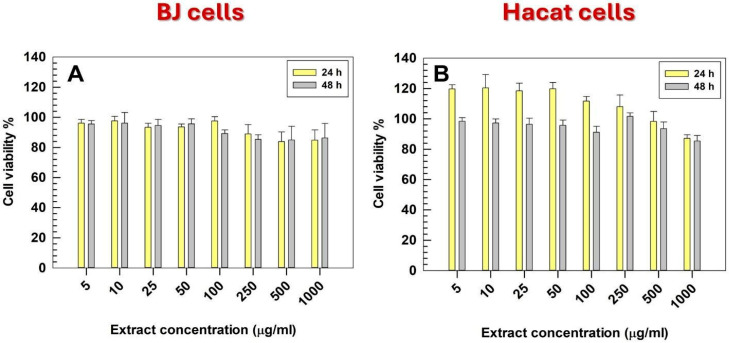
Cell viability. In vitro cell viability assay on human fibroblast (**A**) and human keratinocytes (**B**). The cytocompatibility of SM extract was tested at different concentrations ranging between 5 and 1000 µg/mL. Results are expressed as the means of three independent experiments ± S.D.

The cytocompatibility of the SM aqueous extract was evaluated on two skin-representative cell lines: human keratinocytes (HaCaT) and dermal fibroblasts (BJ) [38]. The extract did not exert cytotoxic effects at any of the tested concentrations, maintaining cell viability consistently above 85% for both cell lines (Figure 2). This high level of biocompatibility demonstrated that the phytocomplex did not interfere with the basal metabolic activity of keratinocytes or fibroblasts, even at the highest doses tested. These results are consistent with previous reports describing the safety profile of SM and support its suitable use for skin and dermal application [23]. The confirmed cytocompatibility represented an essential prerequisite for its incorporation into nanoscale delivery systems, such as ultradeformable liposomes, whose safety and skin tolerability are already well documented in the literature [39].

### 3.3. Scalable Preparation of SM-Ultradeformable Liposomes and Physicochemical Characterization

**Figure 3:**
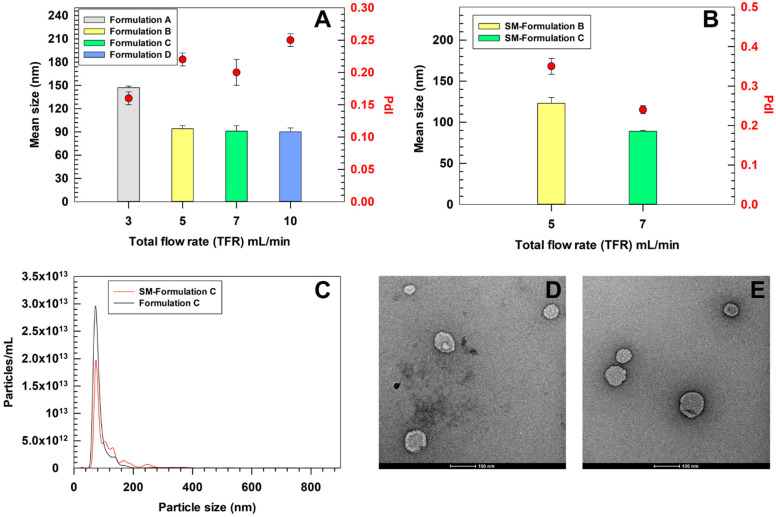
Physicochemical characterization of ultradeformable liposomes. (**A**) Z-Average and PdI of empty formulations obtained by using four different TFR. (**B**) Z-Average and PdI of SM-loaded ultradeformable liposomes obtained at TFR of 5 and 7 mL/min. (**C**) NTA representative curve of size distribution and particle concentration of empty and SM-loaded Formulation C. (**D**) TEM images of empty Formulation C. (**E**) TEM image of SM-loaded Formulation C. Results are the mean of three independent experiments ± S.D. Statistical analysis is reported in the supplementary materials (Table S1).

The microfluidic parameters were optimized to produce ultradeformable liposomes suitable for topical skin application, maintaining the same lipid and surfactant composition previously validated in bulk [31]. The lipid-to-surfactant molar ratio (9:1) and the FRR were kept constant, while the effect of increasing TFR on vesicle size and polydispersity was evaluated by using DLS. In particular, the lipid-to-surfactant molar ratio used in this study was previously tested by our group and shown to be safe on BJ human fibroblast [40]. Similar findings have also been reported in other studies employing different types of surfactants, further supporting the biocompatibility of ultradeformable liposomes [39,41]. An FRR of 3:1 was selected based on condition commonly reported in the literature for microfluidic production of lipid-based nanovesicles [42,43] and recommended in a technical note provided by the Sunshine microfluidic manufacturer (https://www.unchainedlabs.com/wp-content/uploads/2024/04/Sunshine_brochure_RevB.pdf, accessed 1 August 2025). By increasing the TFR, a progressive reduction in mean vesicle size, from 147 ± 2 nm at 3 mL/min to 90 ± 5 nm at 10 mL/min, was recorded (Figure 3). This trend could be explained by the hydrodynamic conditions inside the microfluidic chip, where higher TFRs were associated with smaller liposome size, which may result from more efficient mixing and a shorter residence time that limits vesicle growth and aggregation [44]. At 3 (Formulation A) and 5 mL/min (Formulation B), the calculated Reynolds (Re) numbers, for 490 trident T chip, were ~101 and ~168, respectively, with corresponding Dean (De) numbers of ~50 and ~84, indicating a predominantly laminar regime with moderate secondary Dean vortices. Under these conditions, vesicles formation occurred closer to thermodynamic equilibrium, thus leading to relatively larger but uniform vesicles (PdI 0.16 ± 0.01 and 0.22 ± 0.01, respectively) [45]. At 7 mL/min (Re~236, De~117), the flow regime approached a transitional state where Dean vortices became more pronounced, promoting rapid solvent displacement and faster nucleation of lipid aggregates. This resulted in smaller vesicles (91 ± 7 nm) while maintaining acceptable homogeneity (PdI 0.20 ± 0.02). At 10 mL/min, the hydrodynamic conditions became highly inertial (Re~337, De~167), generating strong Dean vortices and chaotic-like mixing. Although this led to similar vesicles size compared to TFR of 7 mL/min (90 ± 2 nm), the rapid, non-equilibrium assembly at 10 mL/min produced a broader size distribution (PdI 0.25 ± 0.01), likely due to the coexistence of multiple nanovesicle populations formed through distinct nucleation pathways. Similar behaviors were reported for lipid-based nanocarriers produced under extreme mixing conditions, where high shear rates reduced vesicle size but compromised colloidal uniformity [46]. Considering the balance between mean size and PdI, TFRs between 5 and 7 mL/min were found to be optimal for formulating ultradeformable liposomes < 150 nm with PdI ≤ 0.2. Notably, the Sunshine platform by Unchained Labs is specifically designed to support industrial scale-up. Once an optimal formulation is obtained, the same microfluidics chip, hardware, and control software can be used in continuous flow mode to reliably produce large volumes, up to 10 L per day, while preserving key quality features such as size and polydispersity. Furthermore, the optimized parameters obtained with Sunshine can be transferred to large scale system Sunbather (Unchained Labs) with minimal process revalidation (https://www.unchainedlabs.com/wp-content/uploads/2024/04/Sunshine_brochure_RevB.pdf, accessed 1 August 2025).

To incorporate the antioxidant-rich *Stellaria media* extract into ultradeformable liposomes, the extract (10 mg/mL) was used as the aqueous phase in our microfluidic system. We compared the physicochemical properties of the loaded ultradeformable liposomes at two previously identified optimal flow rates (5 and 7 mL/min) to assess the extract’s effects on these parameters. The loading of the extract showed a minimal impact on vesicle size: formulations produced at both 5 (SM-Formulation B) and 7 mL/min (SM-Formulation C) maintaining a mean diameter of 123 ± 7 nm and 89 ± 1 nm, respectively, consistent with results obtained for empty nanovesicles. These data confirmed that the extract did not compromise vesicle self-assembly at these specific flow rates, preserving the structural integrity of resulting SM-loaded ultradeformable liposomes. However, the PdI significantly increased upon extract loading in SM-Formulation B, raising a value of 0.35 ± 0.02. Conversely, SM-Formulation C showed an almost overlapped size distribution of empty nanovesicles produced under the same conditions (0.24 ± 0.02 and 0.20 ± 0.02, respectively). Based on these observations, 7 mL/min was found to be the optimal TFR for producing SM-loaded ultradeformable liposomes. This condition achieved the smallest vesicle size and acceptable homogeneity (PdI < 0.25), thus making the resulting formulation the best option for further evaluations. The size distribution and morphology of ultradeformable liposomes (i.e., Formulation C and SM-Formulation C) produced under the optimized conditions (TFR 7 mL/min) were further investigated by NTA and TEM. NTA measurements showed slightly larger mean sizes compared to DLS, reporting a mean diameter of 100 ± 9 nm for empty and 122 ± 6 nm for SM-Formulation C. Nevertheless, these values are comparable to those obtained by DLS (91 nm and 89 nm, respectively), confirming the overall consistency of the two analytical approaches. The small discrepancies observed could be attributed to the high dilution required for NTA measurements, which reduces the number of particles analyzed, as well as to the different physical principles on which the analyses are based [47]. TEM analysis confirmed the presence of ultradeformable liposomes with diameters ranging from 90 to 120 nm for both empty and SM-Formulation C, consistent with DLS and NTA results. The preservation of size, PdI, and spherical morphology suggested that the incorporation of the *Stellaria media* extract did not affect the self-assembly or structural integrity of the ultradeformable liposomes.

The combined use of complementary (DLS, NTA) and orthogonal (TEM) techniques provided a reliable and accurate characterization of ultradeformable liposomes’ physicochemical features, a crucial prerequisite for process standardization and future industrial scale-up [48]. The zeta potential of optimized formulation was also investigated. By comparing the results obtained for empty Formulation C and SM-Formulation C, a significant variation was recorded, i.e., −21.3 ± 1.6 mV and −38.0 ± 1.5 mV, respectively. These findings suggested that the extract components may interact with the lipid bilayer, thus modifying the exposure of functional groups on the nanovesicles surface, leading to a more negative zeta potential value. Noteworthy, the more negative zeta potential of SM-Formulation C than empty Formulation C represents an advantage, by providing a higher electrostatic repulsion among the nanovesicles which suggested an improved colloidal stability.

### 3.4. Entrapment Efficiency and Kinetics of Release of SM-Ultradeformable Liposomes

**Figure 4:**
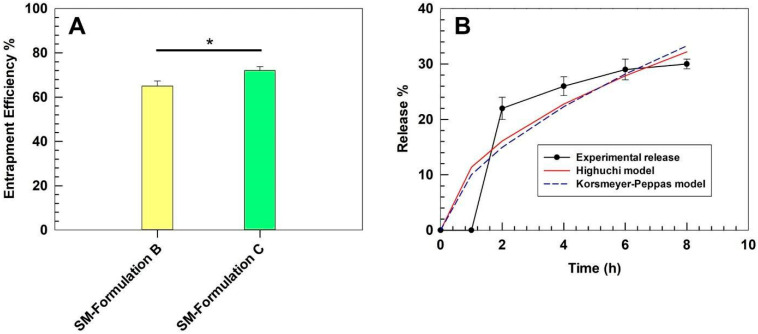
Entrapment efficiency and kinetic release profile. (**A**) showed the entrapment efficiency of SM-Formulation B and SM-Formulation C, while (**B**) reported the kinetic release profile of Formulation C (37 ± 0.5 °C). Results are the means of three independent experiments ± S.D. * *p* < 0.05.

SM-Formulation B and SM-Formulation C were also characterized in terms of entrapment efficiency by comparing the total polyphenols loading to the TPC of the extract solution (10 mg/mL) used during the preparation phase. SM-Formulation C demonstrated a greater EE% (72% ± 1.8) compared to SM-Formulation B (65% ± 2.3) (Figure 4A), which corresponded to a DL% of 51.9% ± 0.62 and 49.4% ± 0.88, respectively. The entrapment efficiencies observed for SM-Formulations B and C are aligned with the physicochemical features. Indeed, SM-Formulation B displayed a significantly larger mean size and a higher polydispersity index than Formulation C, suggesting a less compact and less homogeneous vesicular structure, which may cause the partial leakage of extract during the purification steps, thus resulting in a lower final encapsulation efficiency. Based on the greater entrapment efficiency and the better physicochemical features, SM-Formulation C was used for further investigations.

The in vitro release profile of optimized SM-Formulation C was investigated under sink conditions up to 8 h. A gradual release was observed, with 22% of SM extract released after 2 h, followed by a slower and sustained release phase, reaching 30% at 8 h (Figure 4B). This biphasic kinetic suggested an initial burst phase, likely due to the desorption of weakly surface-associated biomolecules, followed by a slower diffusion-controlled release of entrapped extract within the lipid bilayer. Sustained release behavior is particularly advantageous for topical applications, as it may provide a depot system, thus prolonging the local antioxidant activity at the skin level while minimizing systemic absorption. To further elucidate the release mechanism, the experimental data were fitted to two classical release models: the Higuchi diffusion model and the Korsmeyer–Peppas power law.

The Higuchi model (R^2^ = 0.82) provided a good approximation of the experimental data, with a release constant k ≈ 11.38%·h^−1^/^2^, indicating that the process is predominantly governed by diffusion through the lipid bilayer. The Korsmeyer–Peppas model (R^2^ = 0.71) yielded a release constant k ≈ 10.04 and a release exponent n ≈ 0.58. These values, falling within the range 0.45–0.89, suggested an anomalous (non-Fickian) transport, where the diffusion, which is the primary mechanism, is probably associated with a minor structural relaxation of the vesicular bilayer [49].

A qualitative evaluation of the gradual release of bioactive molecules from the SM-Formulation C was also performed by analyzing the chromatographic profiles obtained at each time point (Figure S3). The comparison of the chromatograms revealed progressive increments in peak intensities over time, consistent with the cumulative release data, thereby confirming the sustained and controlled release behavior of the optimized formulation.

### 3.5. Long-Term Stability Studies

**Figure 5:**
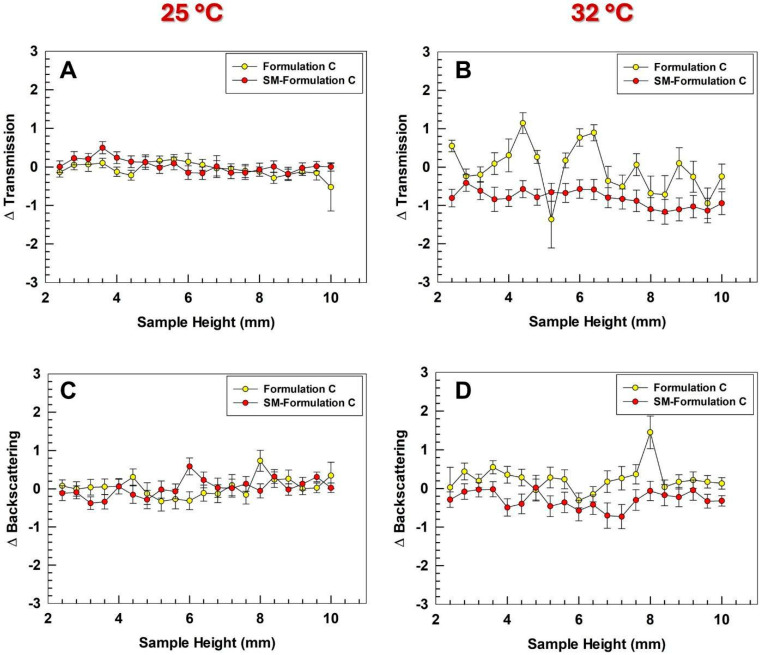
Turbiscan analyses. Variation of transmitted (**A**,**C**) and backscatter (**B**,**D**) light as a function of sample height. The analyses were performed by using Turbiscan Lab instrument at 25 and 32 °C. Results are the mean of three independent analyses ± S.D.

Formulation stability is a critical prerequisite for industrial scale-up, as nanosystems are often prone to colloidal instabilities such as aggregation, sedimentation, or creaming, which can severely limit their large-scale production [50]. To evaluate the long-term stability of the optimized ultradeformable liposomes, Turbiscan lab analyses were performed at room temperature, which is relevant for both manufacturing and storage, and at 32 °C to mimic the skin conditions during the application. The backscattering profiles showed no significant variations, with both ΔT and ΔBS values remaining within the threshold of ±5% at both temperatures (Figure 5). These results demonstrated the absence of destabilization phenomena, confirming that the formulation maintains its colloidal integrity under both conditions, thus supporting its potential for continuous and large-scale manufacturing. The comparison of Turbiscan profiles between empty and extract-loaded ultradeformable liposomes confirmed that the presence of the SM extract did not alter the key physicochemical parameters governing formulation stability. Backscattering and transmission results were also confirmed by Turbiscan stability index (TSI) analysis which further demonstrated similar results for empty and SM-loaded Formulation C (Figure S4), showing kinetic profiles comparable to other ultradeformable liposomes produced in bulk [31]. Despite the comparable data obtained, TSI kinetic analyses revealed a modest enhanced stability of ultradeformable liposomes upon incorporation of the SM extract. This trend is in line with the more negative zeta potential discussed above, which by increasing the electrostatic repulsion may reduce the incidence of destabilization phenomena.

**Figure 6:**
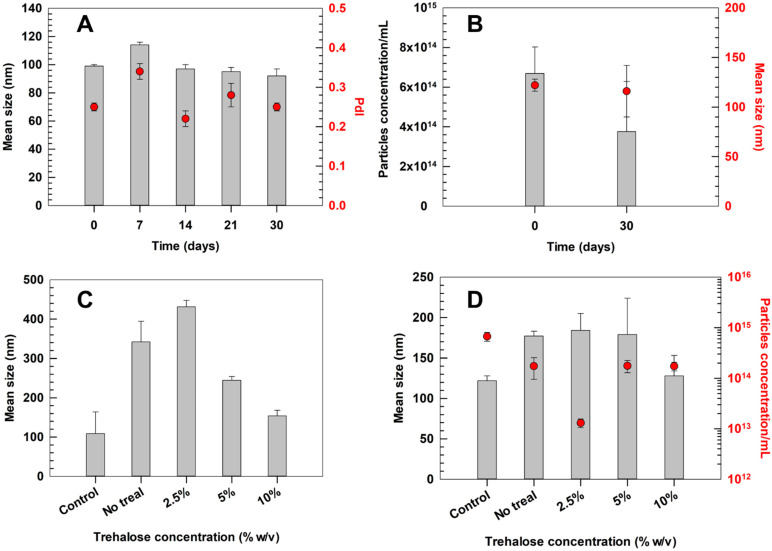
Storage and freeze-drying stability of SM-Formulation C. Stability at +4 °C was studied for up to 30 days and monitored by DLS (**A**) and NTA (**B**). The stability to freeze-drying process was investigated as a function of cryoprotectant concentration. SM-Formulation C was analyzed before (control) and after lyophilization by DLS (**C**) and NTA (**D**). Results are the means of three independent analyses ± S.D. Statistical analysis is reported in Table S2.

To further assess storability, the optimized formulation was monitored over 30 days at 4 °C. DLS analysis revealed no significant changes in mean size that remained stable (ca. 100 nm) with a stability index (SI) of 2% throughout the overall storage time. The PdI fluctuated minimally, corresponding to a SI of 4.5%, indicating a suitable colloidal stability at +4 °C. These findings agreed with NTA analyses, which not only confirmed the mean size measured by DLS after 30 days but also showed no significant variations in particle concentration (Figure 6A,B). The particle integrity index, calculated as the mean of size and particle concentration changes, averaged 4.1%. Each of these values remained below 5–10%, thresholds widely considered acceptable for colloidal stability in pharmaceutical nanocarriers [51].

The stability of the ultradeformable liposomes was further evaluated after lyophilization, as the ability to withstand freeze-drying without losing their physicochemical integrity represents a crucial advantage for industrial scale-up. Indeed, a stable lyophilized product allows an extended shelf-life in dry form while maintaining the expected functionality and structural characteristics upon rehydration [52].

Three concentrations of trehalose (2.5, 5 and 10% *w*/*v*) were tested as cryoprotectants to assess its effectiveness in preserving liposomal integrity during the freeze-drying and rehydration process.

Similar trends were observed for both empty Formulation C (Figure S5) and SM-Formulation C (Figure 6), although this latter appeared intrinsically more stable than the empty one. In the absence of trehalose, the size of SM-Formulation C increased up to ca. 300 nm, which was significantly lower than the mean diameter observed for empty Formulation C under the same conditions (ca. 500 nm). The addition of trehalose progressively improved colloidal stability of SM-Formulation C, showing a concentration-dependent effect. The best results were obtained by using 10% of cryoprotectant, showing a mean size almost overlapped to the pre-lyophilization values and a particle recovery yield which exceeded 2 × 10^14^ particles/mL, demonstrating an effective protection of vesicle integrity during freeze-drying. Similar results were observed for empty Formulation C.

The comparison between the two formulations highlights that extract-loaded ultradeformable liposomes exhibit a slightly higher intrinsic stability to lyophilization compared to empty vesicles, even without a cryoprotectant. This behavior could be attributed to the interaction of the extract’s bioactive components with the lipid bilayer, which may increase bilayer packing and mechanical cohesion, thereby reducing the susceptibility to freeze-induced fusion or rupture [53]. A slight discrepancy was observed between the size measurements carried out by DLS and NTA after lyophilization, with NTA generally reporting values closer to the pre-lyophilization size distribution. These differences can be explained by the distinct analytical principles of the two techniques. DLS estimates a hydrodynamic diameter based on the variation of the light scattering intensity, which is proportional to the sixth power of particle diameter, thus disproportionately affecting the average size even in presence of a small number of large particles or transient aggregates. In contrast, NTA tracks the Brownian motion of individual particles and provides a number-based distribution, making its mean size more representative of the predominant population of intact vesicles. Similar trends have been reported in other studies on lipid-based nanosystems, where NTA offered a more accurate representation of the main population, while DLS is more sensitive to rare aggregation events [54,55]. NTA results were consistent with the particle concentration data, showing a suitable recovery rate in the presence of trehalose, further supporting the preservation of the physicochemical features of ultradeformable liposomes. These results highlighted lyophilization, in the presence of 10% *w*/*v* of trehalose, as a viable strategy for large-scale production and long-term storage of SM-Formulation C. To confirm the integrity of SM-Formulation C under these conditions, the EE% of lyophilized samples were re-quantified, showing a final EE% of 66.2 ± 0.2. These results demonstrated a minimal leakage of the extract during the freeze-drying process in presence of 10% *w*/*v* of trehalose, thus further supporting the effective preservation of main features of SM-Formulation C under these conditions.

### 3.6. Deformability Index of Ultradeformable Liposomes

Deformability is a key parameter for ensuring the cutaneous penetration of liposomes, as it determines their ability to pass through the stratum corneum without rupturing, thus preventing premature cargo release on the skin surface and enabling deeper dermal accumulation. The deformability index was evaluated for the optimized formulation, comparing empty Formulation C with SM-Formulation C. A liposomal formulation, characterized by a more rigid and tightly packed bilayer, was used as a negative control [56].

**Figure 7:**
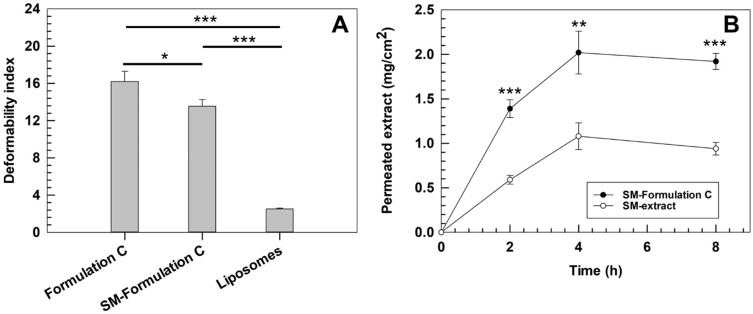
Deformability index (**A**) and percutaneous permeation (**B**) studies. Analyses were performed at 32 ± 0.5 °C and results are reported as the mean of three independent experiments ± S.D. * *p* < 0.05, ** *p* < 0.01, *** *p* < 0.001.

Empty ultradeformable liposomes exhibited a deformability index of 16.2 ± 1.1, approximately 8-fold higher than liposomes (2.5 ± 0.1), confirming their superior ability to undergo reversible shape deformation (Figure 7A). SM-Formulation C displayed a slightly lower deformability index (13.6 ± 0.7) than empty one, which can be attributed to interactions between the extract’s bioactive molecules and the phospholipids of nanovesicles. These results supported the freeze-drying data above discussed and further corroborated the bilayer packing effect of SM extract [57]. Nevertheless, SM-Formulation C retained a deformability index nearly six-fold higher than liposomes demonstrating that the incorporation of the extract does not compromise the overall vesicles deformability. Further confirmation of vesicle structural integrity after mechanical stress was obtained by DLS analysis. Following the deformability test, ultradeformable liposomes exhibited only a minimal size reduction while the polydispersity index remained unchanged. These results proved that the vesicles largely recovered their original structure after being forced through pores approximately one-third of their average size, confirming the absence of significant vesicle disruption or aggregation during the process.

### 3.7. Percutaneous Permeation Studies

The percutaneous permeation study confirmed the capability of optimized ultradeformable liposomes to significantly improve the permeation of loaded extract through SCE membrane. A significant higher permeation of SM-Formulation C than free extract was recorded for all investigated time points. After 8 h, the amount of extract permeated through the SCE membrane was approximately two-fold higher for SM-Formulation C than free extract solution. In detail, SM-Formulation C allowed an extract permeation rate of 1.92 ± 0.09 mg/cm^2^, whereas the permeation extent obtained by using the free extract was only 0.94 ± 0.07 mg/cm^2^ (Figure 7B). The calculated transdermal flux (J) was 0.24 mg/(cm^2^·h) for SM-Formulation C and 0.12 mg/(cm^2^·h) for the free extract, in line with the apparent permeability coefficient (Papp) showed (0.033 cm/h vs. 0.016 cm/h, respectively.

These findings are consistent with the great structural flexibility of ultradeformable liposomes, which facilitates their passage through the narrow intercellular pathways of the stratum corneum, allowing a greater accumulation of payload in the deeper skin layers. Notably, this increased permeation was achieved without compromising vesicle integrity, as confirmed by the minimal variations in size and PdI detected by DLS analysis after the deformability test. The parallel increase in both J and Papp strongly supports the hypothesis that the improved permeation of loaded extract is primarily related to the intrinsic physicochemical properties of the ultradeformable liposomes rather than to concentration-driven effects [58]. Indeed, since Papp normalizes the flux to the initial dose, this trend confirmed that the enhanced delivery resulted from the vesicles’ high deformability and structural resilience, rather than from differences in initial loading or superficial retention.

### 3.8. Skin Tolerability Studies on Healthy Human Volunteers

The safety of the formulation was evaluated in healthy human volunteers using a panel of non-invasive probes selected to monitor complementary aspects of skin physiology relevant to cutaneous tolerability. Skin hydration and colorimetric parameters were used as indicators of cutaneous safety, while transepidermal water loss was assessed as a marker of barrier integrity [59]. This last parameter acquires a crucial role in the study of skin tolerability of ultradeformable liposomes, which are designed to deform and transiently interact with the stratum corneum, thus potentially causing the destabilization of this barrier, leading to measurable reductions in hydration or to an increase in TEWL values [60].

**Figure 8:**
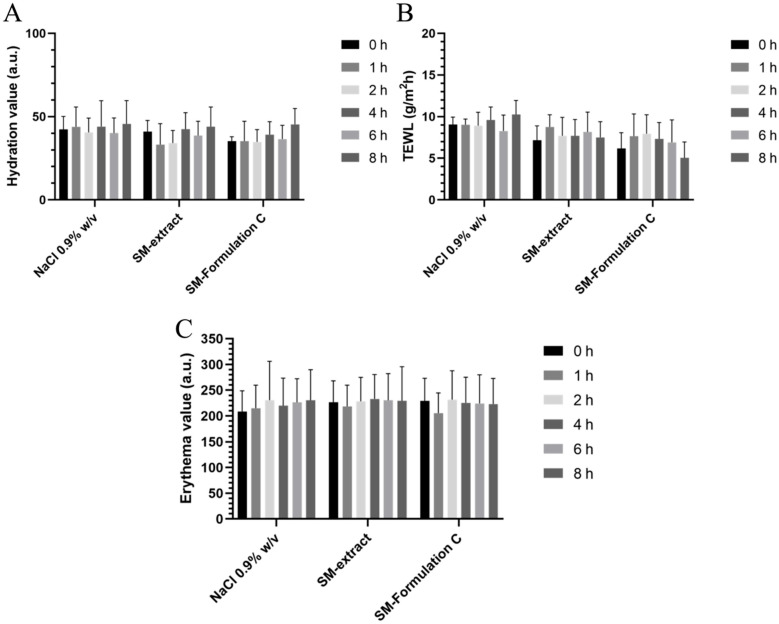
Skin tolerability. Skin hydration (**A**), transepidermal water loss (**B**), and erythema index (**C**) monitored within 8 h after the topical application of formulations. Saline solution was considered as negative control and each time point was compared to the referred baseline value (t_0_) to evaluate any changes. Data are the means of three different measurements on each analyzed site ± SD. (n = 8).

Following topical application of SM-extract and SM-Formulation C, no significant changes in skin hydration were detected (Figure 8A), with results comparable to those obtained after treatment with the saline control (NaCl 0.9% *w*/*v*). These findings suggested that the formulations did not affect the hydration balance of the stratum corneum. Likewise, TEWL values remained stable throughout the overall observation period (Figure 8B), further supporting the suitable tolerability profile of SM-extract and SM-ultradeformable liposomes.

To further exclude potential irritation, skin colorimetric parameters were analyzed using two complementary approaches. The erythema index was first quantified by reflectance spectroscopy (X-Rite Ci62), which provides spectral information across the visible range (Figure 8C), and subsequently confirmed by Mexameter^®^ MX300 measurements (Figure S6), which directly assess hemoglobin- and melanin-dependent absorbance at selected wavelengths [61]. Both methods consistently indicated the absence of changes in erythema and melanin indices within 8 h following the skin application of formulations. Overall, the consistency across hydration, TEWL, and dual-method colorimetric analyses provided strong evidence that the tested formulations were well tolerated and maintained cutaneous homeostasis, supporting their suitability for further development as safe topical delivery systems.

## 4. Conclusions

This work highlighted a scalable and reproducible approach for the development of ultradeformable liposomes using a GMP-like microfluidic platform. By integrating formulation design with process optimization, we developed a robust formulation capable of delivering Stellaria Media’s extract with improved dermal performance. Careful consideration was given to parameters essential for industrial scale-up, such as size control (< 300 nm), colloidal stability during manufacturing and storage, and compatibility with lyophilization. Beyond the technological aspects, the proposed ultradeformable liposomes also demonstrated the ability to enhance the percutaneous permeation of the encapsulated phytocomplex, addressing one of the main limitations associated with the topical use of natural compounds. This feature is remarkably relevant due to the growing interest in polyphenol-rich extracts for pharmaceutical and cosmeceutical application. In these attempts, this study offers a concrete example of how advanced formulation strategies can be combined with scalable processes to meet both functional and quality requirements for skin-targeted delivery.

## Figures and Tables

**Table 1:** Microfluidics parameters. Optimization parameters of the ultradeformable liposomes production.

Formulation	FRR	TFR (μL/min)	Water FR (μL/min)	Organic FR (μL/min)
**A**	3:1	3000	2250	750
**B**	3:1	5000	3750	1250
**C**	3:1	7000	5250	1750
**D**	3:1	10,000	7500	2500

**Table 2:** Chemical characterization of *Stellaria media* extract. Results are expressed as the mean of three independent analyses ± S.D. Statistical significance: *** *p* < 0.001 (Stellaria extract inhibition percentage vs. negative control).

POLYPHENOLS			
Mean ABS 760 nm	Mean GAE (mg/mL) ± SD		Mean GAE (mg/g) ± SD
0.634 ± 0.053	0.131 ± 0.011		13.1 ± 1.1
**ANTIOXIDANT ACTIVITY**			
**ABTS ASSAY**	**Mean ABS 730 nm**	**Mean I** (**%**) **± SD**	**AAE** (**µg**) **±SD**
CTRL (−)	0.1995 ± 0.0064	---	---
CTRL (+)	0.0035 ± 0.0021	~98	---
Stellaria Extract	0.030 ± 0.001 (***)	84.96 ± 0.71 (***)	23.09 ± 0.19
**DPPH ASSAY**	**Mean ABS 517 nm**	**Mean I** (**%**) **± SD**	**AAE** (**µg**) **± SD**
CTRL (−)	1.1696 ± 0.0003	---	---
CTRL (+)	0.0924 ± 0.0016	~92	---
Stellaria Extract	0.8977 ± 0.0017 (***)	23.25 ± 0.14 (***)	12.58 ± 0.09

ABS = absorbance; GAE = gallic acid equivalents; SD = standard deviation; I (%) = inhibition percentage; AAE = ascorbic acid equivalents.

## Data Availability

Dataset available on request from the corresponding.

## Supplementary Materials

The following supporting information can be downloaded at https://www.mdpi.com/article/10.3390/pharmaceutics17111390/s1, Figure S1: Results of the SM extract HPLC analyses; Figure S2: Schematic representation of Sunny Trident T 490 (S490) microfluidic chip (Unchained Labs, USA); Figure S3: HPLC qualitative analyses of release kinetic profile of SM-Formulation C; Figure S4: Turbiscan stability index of optimized formulation; Figure S5: Stability of empty Formulation C to the freeze-drying process as a function of cryoprotectant concentration; Figure S6: Melanin and erythema values in healthy human volunteers; Table S1: Statistical analysis of physicochemical features of ultradeformable liposomes; Table S2: Statistical analysis of SM-Formulation C stored at 4 °C and after lyophilization.

## References

[1] Wagner A., Vorauer-Uhl K. (2011). Liposome technology for industrial purposes. J. Drug Deliv..

[2] Matharoo N., Mohd H., Michniak-Kohn B. (2024). Transferosomes as a transdermal drug delivery system: Dermal kinetics and recent developments. Wiley Interdiscip. Rev. Nanomed. Nanobiotechnol..

[3] Eugster R., Luciani P. (2025). Liposomes: Bridging the gap from lab to pharmaceuticals. Curr. Opin. Colloid Interface Sci..

[4] Crommelin D.J.A., van Hoogevest P., Storm G. (2020). The role of liposomes in clinical nanomedicine development. What Now? Now What?. J. Control. Release.

[5] Boakye C.H.A., Patel K., Doddapaneni R., Bagde A., Behl G., Chowdhury N., Safe S., Singh M. (2016). Ultra-flexible nanocarriers for enhanced topical delivery of a highly lipophilic antioxidative molecule for skin cancer chemoprevention. Colloids Surf. B Biointerfaces.

[6] Cristiano M.C., d’Avanzo N., Mancuso A., Tarsitano M., Barone A., Torella D., Paolino D., Fresta M. (2022). Ammonium Glycyrrhizinate and Bergamot Essential Oil Co-Loaded Ultradeformable Nanocarriers: An Effective Natural Nanomedicine for In Vivo Anti-Inflammatory Topical Therapies. Biomedicines.

[7] Fernández-García R., Lalatsa A., Statts L., Bolás-Fernández F., Ballesteros M.P., Serrano D.R. (2020). Transferosomes as nanocarriers for drugs across the skin: Quality by design from lab to industrial scale. Int. J. Pharm..

[8] Singh A., Fatima Z., Srivastava D. (2025). A Comprehensive Review on Polyphenols based Nanovesicular System for Topical Delivery. Curr. Drug Deliv..

[9] Wu P.S., Li Y.S., Kuo Y.C., Tsai S.J., Lin C.C. (2019). Preparation and Evaluation of Novel Transfersomes Combined with the Natural Antioxidant Resveratrol. Molecules.

[10] Ashtiani H.A., Bishe P., Lashgari N.-A., Nilforoushzadeh M.A., Zare S. (2016). Liposomes in cosmetics. J. Ski. Stem Cell.

[11] Fytianos G., Rahdar A., Kyzas G.Z. (2020). Nanomaterials in Cosmetics: Recent Updates. Nanomaterials.

[12] Bilal M., Iqbal H.M. (2020). New insights on unique features and role of nanostructured materials in cosmetics. Cosmetics.

[13] Figueroa-Robles A., Antunes-Ricardo M., Guajardo-Flores D. (2021). Encapsulation of phenolic compounds with liposomal improvement in the cosmetic industry. Int. J. Pharm..

[14] Halliwell B. (2024). Understanding mechanisms of antioxidant action in health and disease. Nat. Rev. Mol. Cell Biol..

[15] Anik M.I., Mahmud N., Masud A.A., Khan M.I., Islam M.N., Uddin S., Hossain M.K. (2022). Role of Reactive Oxygen Species in Aging and Age-Related Diseases: A Review. ACS Appl. Bio Mater..

[16] Liu T., Zhu S., Yang Y., Qin W., Wang Z., Zhao Z., Liu T., Wang X., Duan T., Liu Y. (2024). Oroxylin A ameliorates ultraviolet radiation-induced premature skin aging by regulating oxidative stress via the Sirt1 pathway. Biomed. Pharmacother..

[17] Tang Z., Liu Z., Zhang Y., Luo S., Xu Y., Ren L. (2024). Functional hyaluronic acid microneedles for skin photoaging based on collagen induction and oxidative stress regulation strategies. Int. J. Biol. Macromol..

[18] Ferreira L., Torres B., Hameed H., Vieira A.C., Singh S.K., Dua K., Veiga F., Pires P.C., Mazzola P.G., Paiva-Santos A.C. (2024). Updated insights of active cosmetic ingredients against blue light: In vivo and in vitro evidence. J. Drug Deliv. Sci. Technol..

[19] Rusu M.E., Fizeșan I., Vlase L., Popa D.S. (2022). Antioxidants in Age-Related Diseases and Anti-Aging Strategies. Antioxidants.

[20] Michalak M. (2022). Plant-Derived Antioxidants: Significance in Skin Health and the Ageing Process. Int. J. Mol. Sci..

[21] Choi H.Y., Lee Y.J., Kim C.M., Lee Y.-M. (2024). Revolutionizing cosmetic ingredients: Harnessing the power of antioxidants, probiotics, plant extracts, and peptides in personal and skin care products. Cosmetics.

[22] Hoang H.T., Moon J.-Y., Lee Y.-C. (2021). Natural antioxidants from plant extracts in skincare cosmetics: Recent applications, challenges and perspectives. Cosmetics.

[23] Miere F., Teușdea A.C., Laslo V., Cavalu S., Fritea L., Dobjanschi L., Zdrinca M., Zdrinca M., Ganea M., Pașc P. (2021). Evaluation of in vitro wound-healing potential, antioxidant capacity, and antimicrobial activity of *Stellaria media* (L.) Vill. Appl. Sci..

[24] Miere F.G., Ganea M., Teodorescu A.G., Horvath T., Hanga-Farcas A., Csaba N., Zdinca M., Zdinca M., Dobjanschi L. (2023). Characterization in terms of phytochemical content and medicinal potential of the *Stellaria media* plant extract. Pharmacophore.

[25] Oladeji O.S., Oyebamiji A.K. (2020). *Stellaria media* (L.) Vill.—A plant with immense therapeutic potentials: Phytochemistry and pharmacology. Heliyon.

[26] Cheng Y.C., Li T.S., Su H.L., Lee P.C., Wang H.D. (2020). Transdermal Delivery Systems of Natural Products Applied to Skin Therapy and Care. Molecules.

[27] Ricci A., Stefanuto L., Gasperi T., Bruni F., Tofani D. (2024). Lipid Nanovesicles for Antioxidant Delivery in Skin: Liposomes, Ufasomes, Ethosomes, and Niosomes. Antioxidants.

[28] Olaru O.T., Nitulescu G.M., Codreanu A.M., Calmuc V.A., Venables L., van de Venter M., Gird C.E., Duta-Bratu C.G., Nitulescu G. (2024). Inhibitory Effects on Staphylococcus aureus Sortase A by Aesculus sp. Extracts and Their Toxicity Evaluation. Plants.

[29] Mare R., Pujia R., Maurotti S., Greco S., Cardamone A., Coppoletta A.R., Bonacci S., Procopio A., Pujia A. (2023). Assessment of Mediterranean Citrus Peel Flavonoids and Their Antioxidant Capacity Using an Innovative UV-Vis Spectrophotometric Approach. Plants.

[30] Settino M., Maurotti S., Tirinato L., Greco S., Coppoletta A.R., Cardamone A., Musolino V., Montalcini T., Pujia A., Mare R. (2024). Zibibbo Grape Seeds’ Polyphenolic Profile: Effects on Bone Turnover and Metabolism. Pharmaceuticals.

[31] Celia C., Cilurzo F., Trapasso E., Cosco D., Fresta M., Paolino D. (2012). Ethosomes^®^ and transfersomes^®^ containing linoleic acid: Physicochemical and technological features of topical drug delivery carriers for the potential treatment of melasma disorders. Biomed. Microdevices.

[32] Adamo G., Fierli D., Romancino D.P., Picciotto S., Barone M.E., Aranyos A., Božič D., Morsbach S., Raccosta S., Stanly C. (2021). Nanoalgosomes: Introducing extracellular vesicles produced by microalgae. J. Extracell. Vesicles.

[33] Palmosi T., Tolomeo A.M., Cirillo C., Sandrin D., Sciro M., Negrisolo S., Todesco M., Caicci F., Santoro M., Dal Lago E. (2022). Small intestinal submucosa-derived extracellular matrix as a heterotopic scaffold for cardiovascular applications. Front. Bioeng. Biotechnol..

[34] Mancuso A., Cristiano M.C., d’Avanzo N., Panza S., Tarsitano M., Celia C., Paolino D., Fresta M. (2025). Assessing effectiveness of multistage nanomedicines for multidrug therapy of vitiligo. J. Drug Deliv. Sci. Technol..

[35] van den Bergh B.A., Bouwstra J.A., Junginger H.E., Wertz P.W. (1999). Elasticity of vesicles affects hairless mouse skin structure and permeability. J. Control. Release.

[36] Bruno M.C., Gagliardi A., Mancuso A., Barone A., Tarsitano M., Cosco D., Cristiano M.C., Fresta M., Paolino D. (2022). Oleic acid-based vesicular nanocarriers for topical delivery of the natural drug thymoquinone: Improvement of anti-inflammatory activity. J. Control. Release.

[37] Gelker M., Müller-Goymann C.C., Viöl W. (2018). Permeabilization of human stratum corneum and full-thickness skin samples by a direct dielectric barrier discharge. Clin. Plasma Med..

[38] Ziemlewska A., Zagórska-Dziok M., Nizioł-Łukaszewska Z. (2021). Assessment of cytotoxicity and antioxidant properties of berry leaves as by-products with potential application in cosmetic and pharmaceutical products. Sci. Rep..

[39] Souto E.B., Macedo A.S., Dias-Ferreira J., Cano A., Zielińska A., Matos C.M. (2021). Elastic and Ultradeformable Liposomes for Transdermal Delivery of Active Pharmaceutical Ingredients (APIs). Int. J. Mol. Sci..

[40] Paolino D., Cosco D., Cilurzo F., Trapasso E., Morittu V.M., Celia C., Fresta M. (2012). Improved in vitro and in vivo collagen biosynthesis by asiaticoside-loaded ultradeformable vesicles. J. Control. Release.

[41] Avadhani K.S., Manikkath J., Tiwari M., Chandrasekhar M., Godavarthi A., Vidya S.M., Hariharapura R.C., Kalthur G., Udupa N., Mutalik S. (2017). Skin delivery of epigallocatechin-3-gallate (EGCG) and hyaluronic acid loaded nano-transfersomes for antioxidant and anti-aging effects in UV radiation induced skin damage. Drug Deliv..

[42] Forbes N., Hussain M.T., Briuglia M.L., Edwards D.P., Horst J.H.T., Szita N., Perrie Y. (2019). Rapid and scale-independent microfluidic manufacture of liposomes entrapping protein incorporating in-line purification and at-line size monitoring. Int. J. Pharm..

[43] Levy E.S., Yu J., Estevez A., Mao J., Liu L., Torres E., Leung D., Yen C.W. (2021). A Systematic Approach for Liposome and Lipodisk Preclinical Formulation Development by Microfluidic Technology. AAPS J..

[44] Liu Y., Yang G., Hui Y., Ranaweera S., Zhao C.X. (2022). Microfluidic Nanoparticles for Drug Delivery. Small.

[45] Zook J.M., Vreeland W.N. (2010). Effects of temperature, acyl chain length, and flow-rate ratio on liposome formation and size in a microfluidic hydrodynamic focusing device. Soft Matter.

[46] Balbino T.A., Aoki N.T., Gasperini A.A., Oliveira C.L., Azzoni A.R., Cavalcanti L.P., de la Torre L.G. (2013). Continuous flow production of cationic liposomes at high lipid concentration in microfluidic devices for gene delivery applications. Chem. Eng. J..

[47] Simon C., Borgos S.E., Calzolai L., Nelson B., Parot J., Petersen E.J., Roesslein M., Xu X., Caputo F. (2023). Orthogonal and complementary measurements of properties of drug products containing nanomaterials. J. Control. Release.

[48] Joyce P., Allen C.J., Alonso M.J., Ashford M., Bradbury M.S., Germain M., Kavallaris M., Langer R., Lammers T., Peracchia M.T. (2024). A translational framework to DELIVER nanomedicines to the clinic. Nat. Nanotechnol..

[49] Wadher K., Trivedi S., Rarokar N., Umekar M. (2024). Development and assessment of rutin loaded transfersomes to improve ex vivo membrane permeability and in vitro efficacy. Hybrid. Adv..

[50] Danaei M., Dehghankhold M., Ataei S., Hasanzadeh Davarani F., Javanmard R., Dokhani A., Khorasani S., Mozafari M.R. (2018). Impact of Particle Size and Polydispersity Index on the Clinical Applications of Lipidic Nanocarrier Systems. Pharmaceutics.

[51] Jakubek Z.J., Chen S., Zaifman J., Tam Y.Y.C., Zou S. (2023). Lipid Nanoparticle and Liposome Reference Materials: Assessment of Size Homogeneity and Long-Term −70 °C and 4 °C Storage Stability. Langmuir.

[52] Jiang H., Wang Y., Xu X., Deng L., Feng L., Han J., Liu W. (2023). Effect of oligosaccharides as lyoprotectants on the stability of curcumin-loaded nanoliposomes during lyophilization. Food Chem..

[53] Popova A.V., Hincha D.K. (2016). Effects of flavonol glycosides on liposome stability during freezing and drying. Biochim. Biophys. Acta (BBA) Biomembr..

[54] Filipe V., Hawe A., Jiskoot W. (2010). Critical evaluation of Nanoparticle Tracking Analysis (NTA) by NanoSight for the measurement of nanoparticles and protein aggregates. Pharm. Res..

[55] Chan M.Y., Dowling Q.M., Sivananthan S.J., Kramer R.M. (2017). Particle Sizing of Nanoparticle Adjuvant Formulations by Dynamic Light Scattering (DLS) and Nanoparticle Tracking Analysis (NTA). Vaccine Adjuvants.

[56] Dayan N., Touitou E. (2000). Carriers for skin delivery of trihexyphenidyl HCl: Ethosomes vs. liposomes. Biomaterials.

[57] Zhang Y., Pu C., Tang W., Wang S., Sun Q. (2020). Effects of four polyphenols loading on the attributes of lipid bilayers. J. Food Eng..

[58] Neupane R., Boddu S.H., Renukuntla J., Babu R.J., Tiwari A.K. (2020). Alternatives to biological skin in permeation studies: Current trends and possibilities. Pharmaceutics.

[59] Schoenfelder H., Liu Y., Lunter D.J. (2023). Systematic investigation of factors, such as the impact of emulsifiers, which influence the measurement of skin barrier integrity by in-vitro trans-epidermal water loss (TEWL). Int. J. Pharm..

[60] Guillot A.J., Martínez-Navarrete M., Garrigues T.M., Melero A. (2023). Skin drug delivery using lipid vesicles: A starting guideline for their development. J. Control. Release.

[61] Vasudevan S., Vogt W.C., Weininger S., Pfefer T.J. (2024). Melanometry for objective evaluation of skin pigmentation in pulse oximetry studies. Commun. Med..

