# Supplementary material for: Microfluidic Design of Ultradeformable Liposomes for Advanced Skin Delivery of Stellaria media Phytocomplex
Source: Pharmaceutics. 2025 Oct 27;17(11):1390. doi: 10.3390/pharmaceutics17111390 (PMC12655562; doi:10.3390/pharmaceutics17111390)
Supplement: Supplementary file 1 [file pharmaceutics-17-01390-s001.zip › pharmaceutics-3835651-supplementary.pdf]

Microfluidic Design of Ultradeformable Liposomes for Advanced Skin Delivery of Stellaria media Phytocomplex

Luigi Ciriolo, Nicola d'Avanzo, Antonia Mancuso, Maria Chiara Cristiano, Antonella Barone, Rosario Mare, Anna Maria Tolomeo, Alexandra I. Comaniciu, Georgiana Nitulescu, Octavian Tudorel Olaru, Felisa Cilurzo, Donatella Paolino and Massimo Fresta

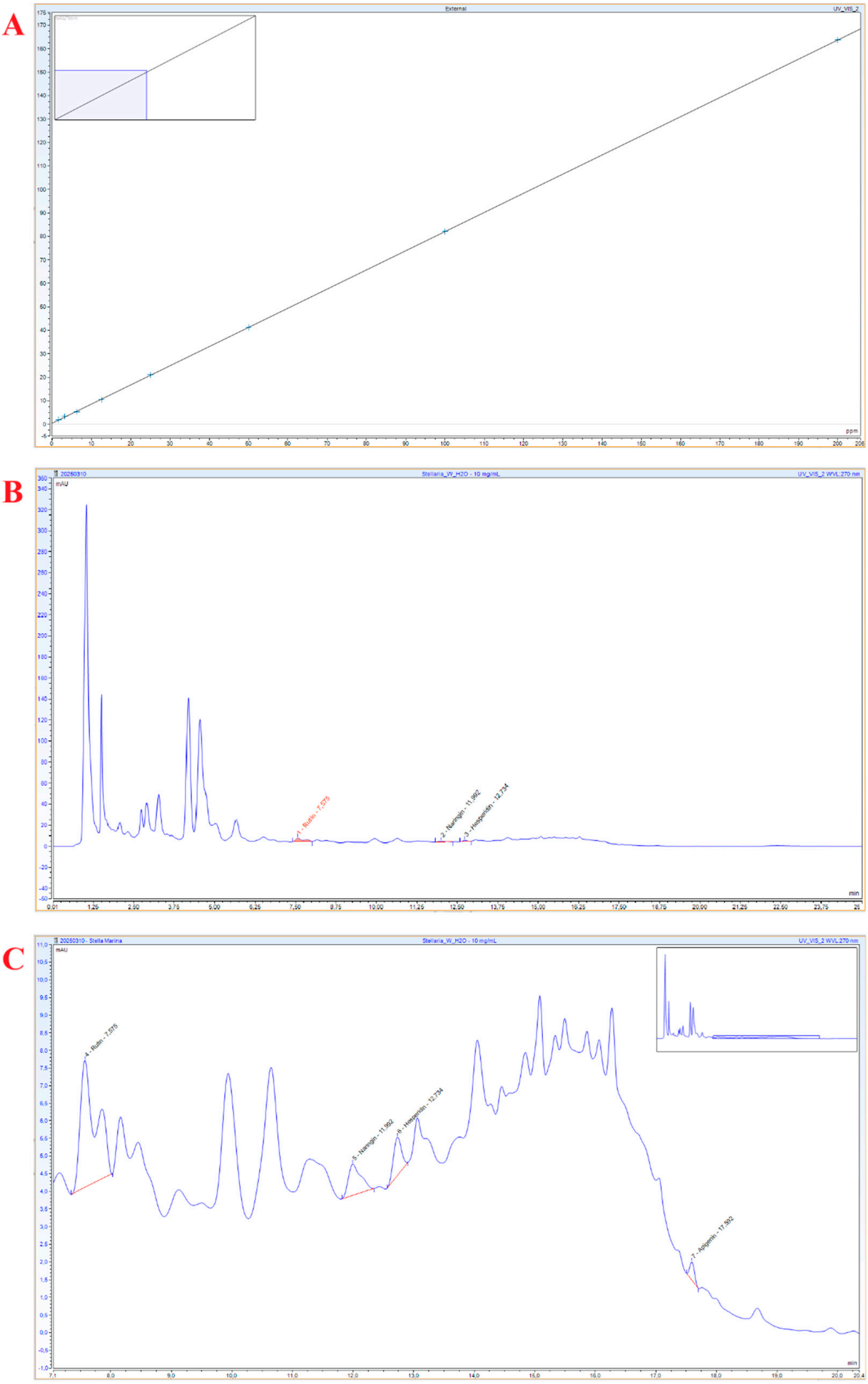

Figure S1. Results of the SM extract HPLC analyses. Panel A shows a representative calibration curve generated using standard reference compounds. Panel B presents the chromatogram of Stellaria media extract (10 mg/mL in water), while Panel C provides a high-resolution view of the flavonoid peaks identified in the extract.

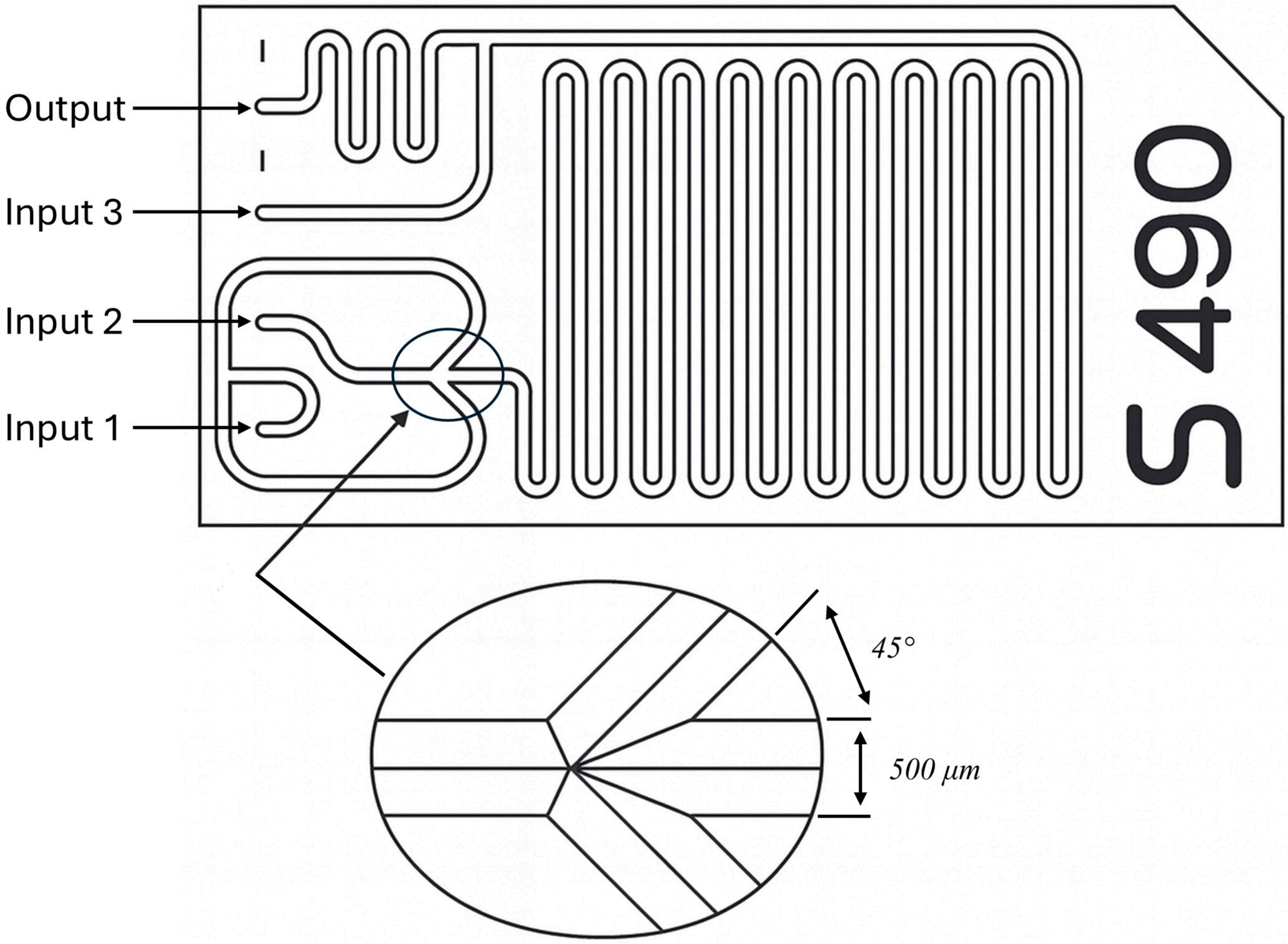

Figure S2. Schematic representation of Sunny Trident T 490 (S490) microfluidic chip (Unchained Labs, USA)

Table S1. Statistical analysis of physicochemical features of ultradeformable liposomes. Comparison of physicochemical features of ultradeformable liposomes realized by using different microfluidic total flow rate. Statistical significance: *p< 0.05, **p< 0.01, and ***p< 0.001.

**Empty Formulations**			
	Mean size	PdI	
Form A vs Form B	***	**	
Form A vs Form C	***	*	
Form A vs Form D	***	***	
Form B vs Form C	N.S.	N.S.	
Form B vs Form D	N.S.	*	
Form C vs Form D	N.S.	*	
**SM-loaded Formulations**			
	Mean size	PdI	ζ
SM-Form B vs Form B	**	***	/
SM-Form C vs Form C	N.S.	N.S.	***
SM-Form B vs SM-Form C	**	**	/

Footnotes: N.S.: no significant; Form: Formulation; SM-Form: Stellaria media-loaded formulation; ζ: zeta potential

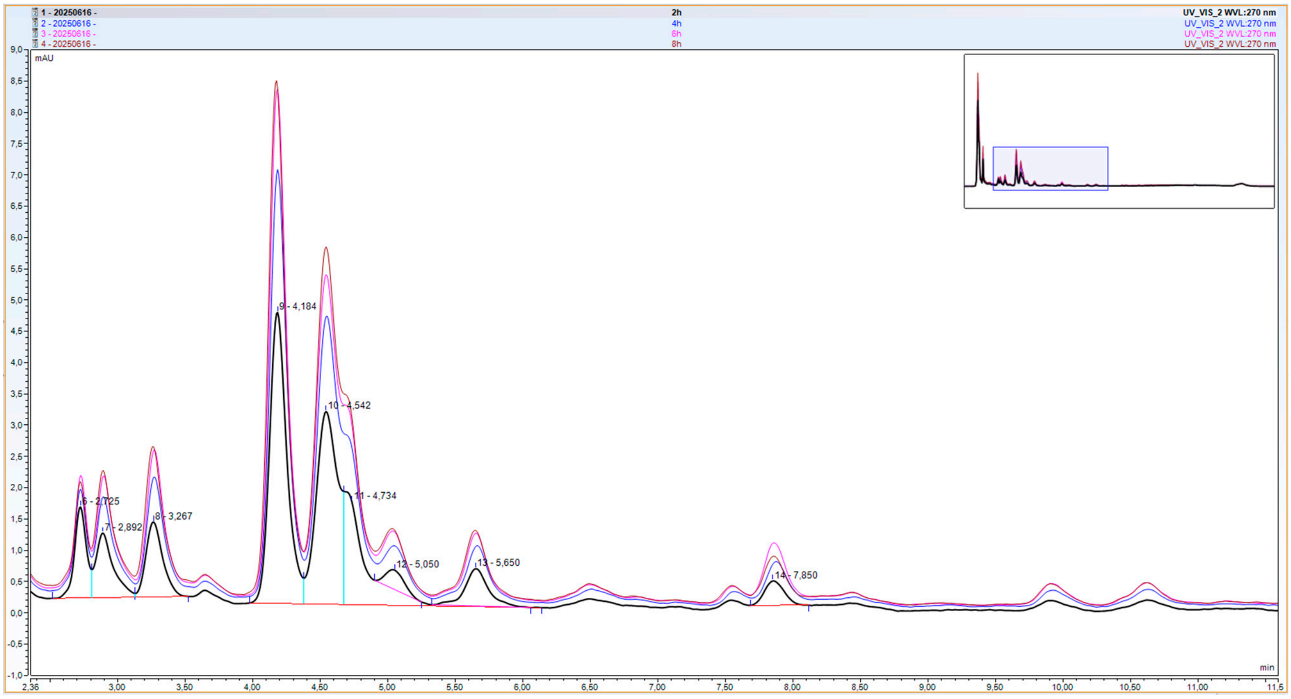

Figure S3. HPLC qualitative analyses of release kinetic profile of SM-Formulation C. HPLC qualitative analysis of aqueous receptor medium used for the evaluation of the release kinetic profile (after 2, 4, 6, and 8 hours) of SM-Formulation C.

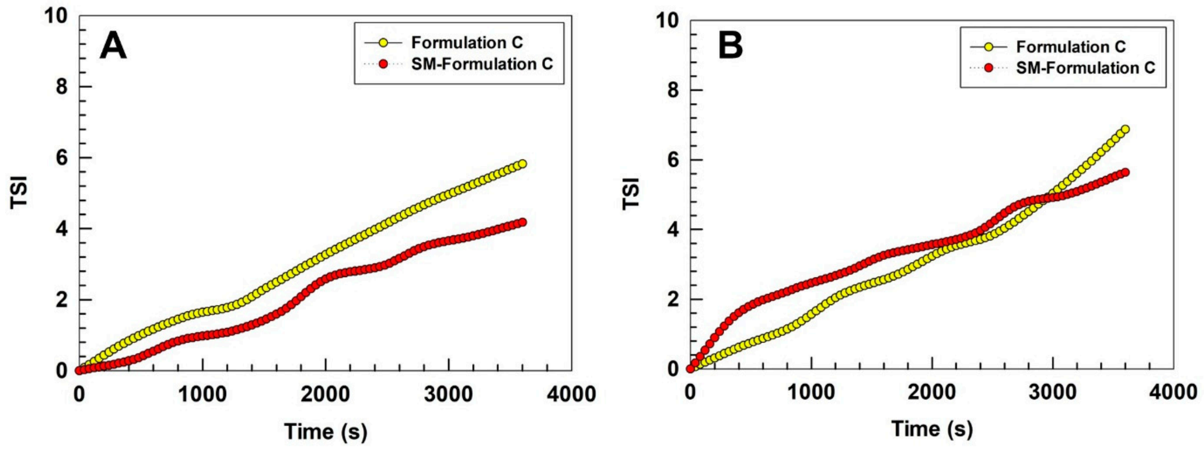

Figure S4. Turbiscan stability index of optimized formulation. Turbiscan stability index (TSI) of Formulation C and SM-Formulation C at 25 °C (Panel A) and 32 °C (Panel B) as a function of time (0–1h). Results are representative of three independent experiments.

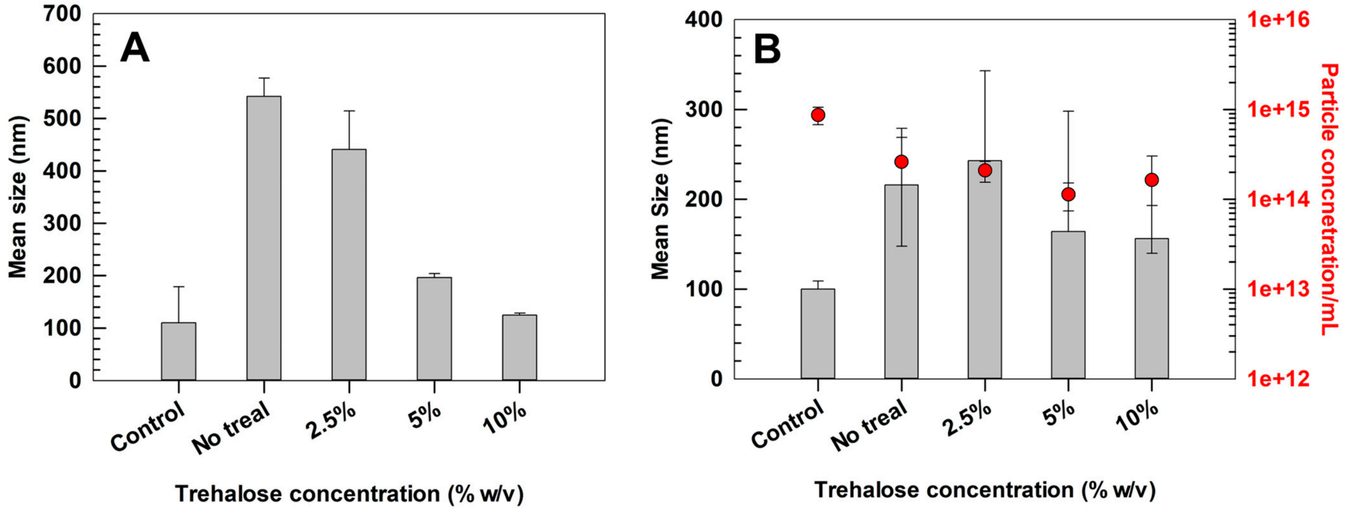

Figure S5. Stability of empty Formulation C to the freeze-drying process. Samples were analyzed before (Control) and after lyophilization by DLS (Panel A) and NTA (Panel B). Results are the mean of three independent analyses ± standard deviation.

Table S2. Statistical analysis of SM-Formulation C stored at 4 °C and after lyophilization. Statistical significance: *p<0.05, **p<0.01 and ***p<0.001.

**Stability at 4 °C**				
	**DLS analysis**		**NTA analysis**	
	Mean size	PdI	Mean size	Particle concentration
t_0 vs 7 days	***	**	/	/
t_0 vs 14 days	N.S.	N.S.	/	/
t_0 vs 21 days	N.S.	N.S.	/	/
t_0 vs 30 days	N.S.	N.S.	N.S.	N.S.
**Stability to freeze-drying**				
	**DSL analysis**		**NTA analysis**	
	Mean size		Mean size	Particle concentration
pre lio vs no trehalose	**		***	**
pre lio vs trehalose 2.5%	***		**	**
pre lio vs trehalose 5%	*		N.S.	**
pre lio vs trehalose 10%	N.S.		N.S.	**

Footnotes: N.S.: no significant

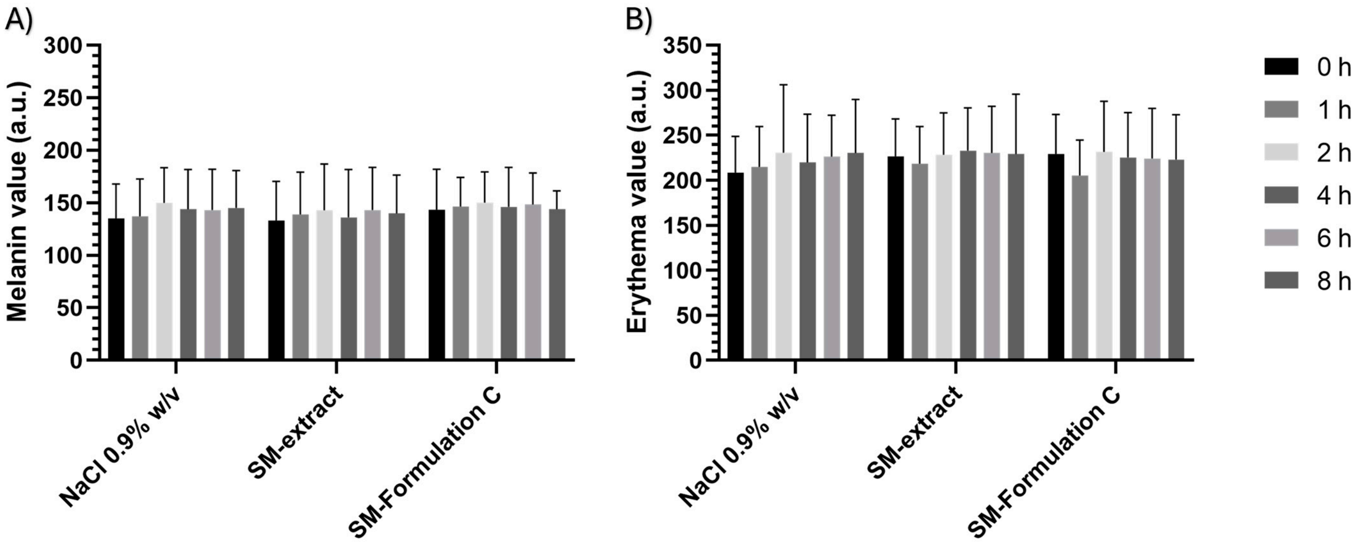

Figure S6. Melanin and erythema values in healthy human volunteers. Variation in melanin (A) and erythema (B) values recorded by using Mexameter MX300. Saline solution was considered as negative control and each time point was compared to the referred baseline value (t0) to evaluate any changes. Data are the mean of three different measurements on each analyzed site ± SD (n=8).
